# Multiple morphological phenotypes of monoclonal immunoglobulin disease on renal biopsy: Significance of treatment 

**DOI:** 10.5414/CNCS110052

**Published:** 2020-04-17

**Authors:** Sreedhar Adapa, Venu Madhav Konala, Srikanth Naramala, Cynthia C. Nast

**Affiliations:** 1 Division of Nephrology, The Nephrology Group, Fresno, CA,; 2 Department of Internal Medicine, Division of Medical Oncology, Ashland Bellefonte Cancer Center, Ashland, KY,; 3 Division of Rheumatology, Adventist Medical Center, Hanford, and; 4 Department of Pathology, Cedars-Sinai Medical Center, Los Angeles, CA, USA

**Keywords:** light chain cast nephropathy (LCCN), AL amyloidosis, monoclonal immunoglobulin deposition disease (MIDD), monoclonal fibrillary glomerulonephritis, proximal tubulopathy

## Abstract

Plasma cell dyscrasias frequently involve the kidney causing renal dysfunction. Multiple morphologic manifestations of κ light chain disease occurring simultaneously in the same kidney biopsy are uncommon and suggest local microenvironment effects in addition to structural properties of the light chain. A 61-year-old female presented with new onset renal failure and proteinuria. Serological workup revealed monoclonal gammopathy with elevated κ : λ ratio of 1,371. Renal biopsy revealed several paraprotein manifestations including κ light chain deposition disease, monoclonal fibrillary glomerulonephritis, cryocrystalglobulenemia and fibrillar/microtubular cast nephropathy. There was also incidental leukocyte chemotactic factor 2 amyloidosis (ALECT 2), negative for κ light chain and confirmed by immunohistochemistry (IHC). Bone marrow biopsy revealed 10 – 20% κ restricted plasma cells. The patient received 10 cycles of CyBorD (cyclophosphamide, bortezomib, and dexamethasone) chemotherapy. Renal function improved with decreased κ : λ ratio. Repeat bone marrow biopsy showed no evidence of abnormal plasma cells by IHC. The renal recovery demonstrates there may be response to chemotherapy irrespective of the morphologic manifestations of light chain-related injury. Additionally, if amyloid is not demonstrated to be of light chain origin, other amyloid types should be considered.

## Introduction 

Paraproteinemias are characterized by clonal proliferation of B or plasma cells resulting in overproduction of a monoclonal protein, which can cause significant renal dysfunction [1]. Monoclonal proteins can induce several morphologic forms of renal injury depending on the local microenvironment and physiochemical properties of the pathologic protein [1]. It is uncommon to encounter more than two forms of light chain injury in the same kidney biopsy [2]. Monoclonal immunoglobulin-induced renal disease may occur with or without associated malignancy, the latter now termed monoclonal gammopathy of renal significance (MGRS) [3]. We report a case of κ light chain myeloma associated with protean manifestations of injury concurrently in a kidney biopsy with excellent response to treatment. 

## Case presentation 

### Clinical history and initial laboratory data 

A 61-year-old female presented with fatigue, dyspnea of 3 months duration, intermittent episodes of epistaxis, and anemia. Her only medication was iron for worsening anemia. Physical examination was significant only for pallor. Initial laboratory is data summarized in Table 1. Urine analysis showed large blood and more than 185 RBC’s on microscopy. 

### Additional investigations 

Serum protein electrophoresis showed atypical gamma fraction with an M-spike of 1.9 mg/dL identified as κ light chain by immunofixation. Urine protein electrophoresis with immunofixation showed κ light chain (67% of paraprotein). Free light chain assessment revealed a κ (15,770) : λ (11.5) light chain ratio of 1,371. Levels of immunoglobulins G, A, and M were either low or within normal limits. ANA (antinuclear antibody), anti-ds-DNA (anti-double stranded DNA antibody), ANCA (anti-neutrophil cytoplasmic autoantibody), anti-GBM (anti–glomerular basement membrane) antibody, and acute hepatitis B and C serologic studies were all negative. The C3 level was low at 31.1 mg/dL, C4 was normal, and Factor H autoantibody was increased at 18.5% (0 – 7.3%). PET/CT (positron emission tomography-computed tomography) scan revealed lytic lesion in the left and right iliac bones and the left femoral diaphysis. A kidney biopsy was performed for worsening renal function and nephrotic range proteinuria. 

### Kidney biopsy 

**Figure 1.**
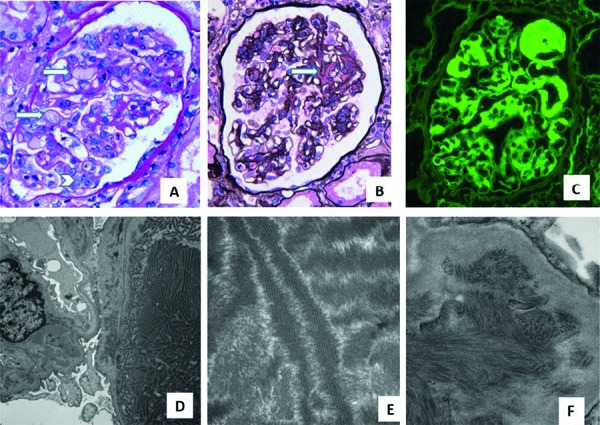
Kidney biopsy showing glomeruli with multiple manifestations of κ light chain injury. A: Glomerulus showing segmental endocapillary PAS-negative thrombi (arrows) and segmental endocapillary inflammation (arrowhead) (periodic acid Schiff (PAS), original magnification × 400). B: Glomerulus with segmental mesangial expansion due to silver-negative material (arrow) (periodic acid-methenamine silver, original magnification × 400). C: Immunofluorescence performed on pronase-digested formalin-fixed paraffin-embedded sections showing κ light chain in glomerular capillary lumina and mesangial regions (original magnification × 400). D: Endocapillary accumulation of wide fibrils (arrow) and mesangial infiltration with smaller electron-dense fibrils (arrowhead) (original magnification × 10,000). E: Endocapillary crystalline fibrils averaging 112 nm with a 22 nm periodicity (original magnification × 72,000). F: Mesangial fibrils averaging 22 nm (original magnification × 29,000).

**Figure 2.**
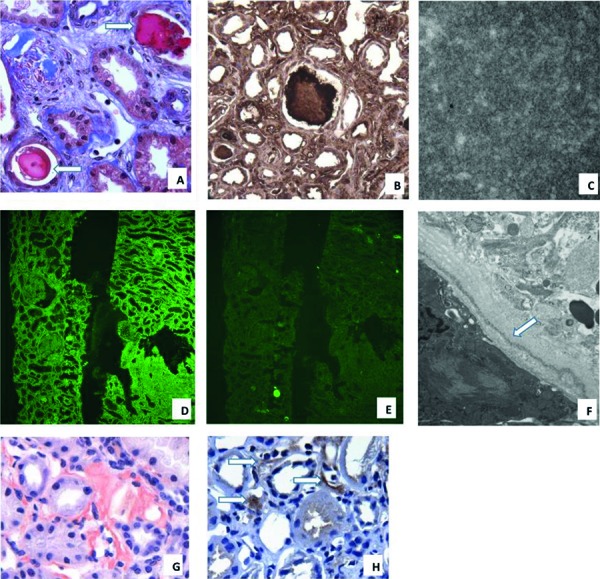
A: Proteinaceous fuchsin-positive dense tubular casts (arrows), Masson’s trichrome, original magnification × 400). B: Strong immunohistochemical staining for κ light chain in a tubular cast (arrow) and in extracellular and basement membrane material (original magnification × 400). C: Tubular cast composed of fibrils and microtubules, averaging 17.7 nm (original magnification × 40,000). D: Immunofluorescence showing linear κ light chain in all extracellular and basement membrane material (original magnification × 200). E: No λ light chain staining by immunofluorescence (original magnification × 200). F: Powdery to finely granular electron-dense material within a tubular basement membrane (arrow) (original magnification × 19,000). G: Positive Congo red stain in interstitial amyloid (original magnification × 200). H: Immunohistochemistry for leukocyte chemotactic factor 2 (LECT2) (arrows) in interstitial Congo red-positive infiltrates (original magnification × 200).

There were 27 glomeruli, 5 of which were globally sclerotic. Light microscopy demonstrated segmental to global endocapillary hypercellularity without crescent formation. (Figure 1) One-third of the glomeruli had segmental periodic acid-Schiff (PAS)-negative, fuchsin-positive (on Masson’s trichrome stain) plasma protein thrombi, and similar material was in the subendothelium and lumens of arterioles and small arteries (Figure 1). 20% of the glomeruli had segmental mesangial expansion due to silver-negative and Congo red-negative material. There was mild tubulointerstitial scarring, and the interstitium was often mildly expanded with Congo red-positive amorphous material that exhibited apple green birefringence with polarized optics (Figure 2). Focally, PAS-negative, fuchsin-positive tubular casts were present with few fractures but no surrounding giant cells. Immunohistochemistry (IHC) showed strong staining of tubular casts and proteinaceous glomerular and vascular thrombi for κ light chain with negative staining for λ light chain in the glomeruli and extracellular material and weak staining of tubular casts. The interstitial Congo red-positive material was negative for κ, λ, AA amyloid, and transthyretin but positive for leukocytic chemotactic factor-2 (LECT2). 

Routine immunofluorescence showed linear staining of all renal basement membranes and extracellular material for κ light chain (3+) and IgG (trace to 1+) (Figure 2). There was segmental staining of glomerular capillary walls and thrombi, and mesangial regions for C3 (2 – 4+). Immunofluorescence on pronase-digested paraffin-embedded sections showed segmental mesangial, and glomerular capillary and vascular thrombi staining for κ light chain (3 – 4+) with negative staining for heavy chains and λ light chain. There was no staining of tubular casts or the interstitium for either light chain by routine or pronase immunofluorescence, although staining for κ light chain was present by IHC. 

Electron microscopy showed powdery electron-dense material in glomerular and tubular basement membranes. Segmentally, mesangial regions contained non-branching fibrils ranging from 16 to 25 nm. Glomerular capillary thrombi were composed of organized crystals 100 – 130 nm in width with an internal periodicity of 22 nm. 10-nm amyloid-type fibrils were present focally in the interstitium but not in glomeruli. Focally, tubular casts were composed of 12 – 21 nm fibrils and microtubules. 

### Diagnosis 

Monoclonal κ light chain-induced renal disease including light chain deposition disease, fibrillar/microtubular light chain cast nephropathy, monoclonal fibrillary glomerulonephritis, and cryocrystalglobulinemia as well as leukocyte chemotactic factor-2 (LECT2) amyloidosis. 

### Clinical follow-up 

Bone marrow biopsy revealed 10 – 20% κ restricted plasma cells, with negative Congo red staining, confirming myeloma. She received 10 cycles of CyBorD (cyclophosphamide, bortezomib, and dexamethasone) chemotherapy, which was well tolerated apart from peripheral neuropathy requiring a dose reduction in bortezomib. Renal function improved with the serum creatinine decreasing to 1.1 mg/dL, and a marked reduction in the κ : λ light chain ratio to 1.7. Repeat bone marrow biopsy showed no evidence of abnormal plasma cells by IHC. Following stem cell mobilization and high dose chemotherapy, the patient received an autologous stem cell transplant and is still in remission 12 months later. 

## Discussion 

Renal involvement occurs in 50% of patients with multiple myeloma and is an independent predictor of poor patient survival [4]. In those with a monoclonal immunoglobulin disorder without malignancy, up to 40% will have MGRS [5]. The circulating pathogenic monoclonal immunoglobulins, which are usually light chains, can cause a variety of renal lesions resulting in glomerular diseases, tubulopathies, renal infiltrates, and casts with obstructive nephropathy [1]. The most frequent manifestation of monoclonal immunoglobulin-induced injury in the kidney is light chain (Bence Jones) cast nephropathy (LCCN), followed by AL amyloidosis and monoclonal (usually light chain) immunoglobulin deposition disease (MIDD) [6]. 

It is uncommon to have more than one, and vanishingly rare to have more than three, concurrent monoclonal immunoglobulin-related lesions in the kidney. Mixed morphologies have included LCCN with MIDD, AL amyloidosis, or proximal light chain crystalline tubulopathy; LCCN with AL amyloidosis and MIDD; and LCCN with fibrils suggestive of fibrillary glomerulonephritis [2, 7, 8, 9, 10]. The reported mixed morphologies of monoclonal immunoglobulin-related renal lesions summarized in Table 2. This likely is due to the complex interactions and effects of monoclonal immunoglobulin handling by different renal cells in the microenvironment, related to inflammatory and cytotoxic reactions, and light chain processing by mesangial cells. Other factors include the physicochemical properties of the abnormal protein such as the amino acid composition of the light chain CDR3 domain allowing binding to Tamm-Horsfall protein to form casts and protein conformation leading to binding-site exposure, the monoclonal immunoglobulin load, and other mechanisms such as inhibition of Factor H activity [6, 11, 12]. Therefore, an unusual combination of the above factors, likely with a prominent effect of the local microenvironment, would need to be present to have diverse intrarenal morphologic expressions of a monoclonal immunoglobulin. 

In the case reported herein, there were κ light chain casts with the staining characteristics of LCCN but demonstrating a fibrillar/microtubular substructure by electron microscopy. Light chain casts typically have a homogeneous granular ultrastructural appearance and may be admixed with Tamm-Horsfall, which is composed of 4-nm fibrils, smaller than amyloid fibrils. When the light chain is λ, infrequently there may be precipitation of amyloid fibrils within the casts, which may occur without systemic amyloid deposition [13, 14]. However, there are no reports of casts containing fibrils and small microtubules, the former similar to those observed in fibrillary glomerulonephritis. This is a unique feature of this biopsy, demonstrating the unusual and protean appearances abnormal κ light chains can display. 

LCCN, MIDD, and fibrillary glomerulonephritis have been reported co-occurring in a renal biopsy [8] but not with cryocrystalglobulinemia. This is an extremely rare complication of monoclonal gammopathy, and has been associated with multiple myeloma, LCCN, and proximal tubulopathy [15]. The mechanism of injury is monoclonal protein, usually IgG heavy chain and κ light chain, precipitation and assembly into crystalline arrays typically depositing in the walls of blood vessels. In patients with cryocrystalglobulinemia, testing for serum cryoglobulins is often negative [15, 16] unlike our patient, who tested positive for cyroglobulins. Renal involvement is more frequent when there are clinical manifestations of skin ulcers, petechiae, purpura, and polyarthralgia, although intraglomerular pseudothrombi, typical for cyroglobulins, may or may not occur [17, 18]. Patients with cryocrystalglobulinemia typically have a poor prognosis, but may respond to treatment as did our patient [18]. 

The patient reported herein had a low C3 level, increased Factor H autoantibody activity, and C3-dominant staining of glomerular thrombi and fibrillary deposits on renal biopsy routine immunofluorescence. Pronase digestion of formalin-fixed paraffin-embedded tissue was necessary to reveal the κ light chain composition of these deposits. There is evidence of monoclonal protein involvement in the activation of the alternative complement pathway, with a λ light chain reported to have Factor H autoantibody activity [11]. This may result in dominant C3 staining on immunofluorescence with associated features of membranoproliferative or C3 glomerulonephritis; therefore, it is important to be aware that these glomerular lesions may be due to an underlying monoclonal gammopathy [19, 20, 21]. The Factor H autoantibody level was high in our patient, potentially resulting in a C3 glomerulonephritis appearance on routine immunofluorescence. This highlights the need for antigen retrieval immunofluorescence on paraffin-embedded kidney tissue in adults with C3 glomerulonephritis to expose masked monotypic proteins. It also demonstrates the value of electron microscopy in identifying unexpected glomerular lesions. 

Another unexpected aspect of this case was the findings of LECT2 amyloidosis in a patient with a monoclonal gammopathy. LECT2, a 16-kD cytokine first isolated by Yamagoe et al. [22] in 1996, is involved in chemotaxis, inflammation, damage, repair, and immunomodulation, and is synthesized primarily in the liver [22]. LECT2 amyloidosis has strong ethnic predisposition involving Hispanics of Mexican decent, like our patient. Other affected ethnic groups include Punjabis, Egyptians, Israelis, and Native Americans, who also are susceptible to LECT 2 amyloidosis [23]. Amyloidosis may be associated with wild-type or mutant LECT2 protein, and has a predilection for the renal cortical interstitium with minor glomerular deposition. This is in contrast to AL amyloid, which usually involves all renal compartments. LECT2 amyloidosis has been found to occur with other renal diseases in one third of cases, most frequently seen with diabetic nephropathy [24]. It is important to bear in mind that a patient with a monoclonal gammopathy may have unrelated renal lesions, and that correct diagnosis is required for appropriate treatment [24]. Therefore, the type of amyloid should always be confirmed by IHC and/or mass spectrometry, particularly if the pattern of amyloid deposition is unexpected or inconsistent with the suspected amyloid type. 

In monoclonal immunoglobulin-associated renal disease, there are histologic features that may suggest a more guarded prognosis including numerous casts in LCCN and cryocrystalgloblins [7, 18]. Regardless, patients with myeloma-related renal insufficiency should receive bortezomib-based chemotherapy with high-dose dexamethasone as soon as possible to decrease light chain production [25]. Lenalidomide is typically avoided in patients with acute kidney injury unless the patient is refractory to other options. Once stabilized, hematopoietic cell transplantation should be considered for eligible patients irrespective of the stage of kidney disease, including patients on dialysis [26]. In addition to the chemotherapy regimen, plasma exchange may help with rapid improvement of symptoms in cryocrystalglobulinemia, with multiple sessions often required [18]. 

In summary, the finding of protean concurrent morphologic manifestations of a monoclonal gammopathy in one biopsy suggests effects of the local microenvironment in addition to structural properties of the light chain. C3 glomerulonephritis in an adult may mask a monoclonal immunoglobulin-related glomerular disease, and the presence of Factor H autoantibody activity does not preclude a diagnosis of monoclonal gammopathy. In this setting, immunofluorescence on formalin-fixed paraffin-embedded tissue after antigen retrieval, IHC for light and heavy chains, and electron microscopy may uncover an underlying monoclonal immunoglobulin or other glomerular lesions. Additionally, in the setting of a monoclonal immunoglobulin, amyloid may not always be light chain-related, and other amyloid types should be considered and evaluated when light chain staining is discrepant to ensure appropriate treatment. Most importantly, even with a spectrum of monoclonal immunoglobulin-related renal findings, chemotherapy may be effective in producing a remission. 

## Key teaching points

- The finding of several concurrent morphologic manifestations of light chain deposition in one biopsy suggests local microenvironment effects in addition to structural properties of the light chain.
- C3 glomerulonephritis (C3GN) in the setting of monoclonal gammopathy may require unmasking by pronase immunofluorescence to identify monoclonal immunoglobulin-related glomerular disease, and EM is additive as it may reveal an unexpected glomerular lesion.
- Factor H autoantibody production may be a mechanism of alternate complement pathway activation in monoclonal gammopathy.
- Amyloid may not always be light chain related, and other amyloid types should be considered and evaluated when light chain staining is discrepant to avoid harmful chemotherapy.
- There may be a renal response to chemotherapy irrespective of the form(s) of light chain deposition in a kidney biopsy.

## Ethics approval 

Our institution does not require ethical approval for reporting individual cases or case series. 

## Funding 

The authors received no financial support for the research, authorship, and/or publication of this article. 

## Conflict of interest 

The authors declare no potential conflicts of interest with respect to the research, authorship, and/or publication of this article. 

**Table 1.** Pertinent laboratory data.

Laboratory test	Initial presentation	Last clinic visit	Reference range
Hemoglobin	7.1 gm/dL	12.1 gm/dL	12 – 16
White blood cell count	5,570 mm^3^	7,900 mm^3^	4,000 – 11,100
Platelet count	150,000	175,000	130,000 – 400,000
Serum creatinine	2.38 mg/dL	1.1 mg/dL	0.5 – 1.2
Serum albumin	3.4 gm/dL	4.2 gm/dL	3.5 – 5.7
Spot urine protein to creatinine ratio	4.3 g/g	Not done	
M-spike	1.9	Not observed	Not observed
κ	15,770 mg/dL	3.7 mg/dL	0.33 – 1.94
λ	11.5 mg/dL	1.8 mg/dL	0.57 – 2.63
κ/λ light chain ratio	1,371	2.04	0.26 – 1.65

**Table 2.** Reported mixed morphologies of monoclonal immunoglobulin-related renal lesions*.

LCCN with MIDD [10]
LCCN, AL amyloidosis [27]
LCCN, AL amyloidosis, MIDD [2]
LCCN, MIDD, fibrillary glomerulonephritis [8]
LCCN, proximal tubulopathy [9]
AL amyloidosis, MIDD [28]
AL amyloidosis, monoclonal fibrillary glomerulonephritis [29]
MIDD, fibrillary glomerulonephritis [30]

LCCN = light chain cast nephropathy; MIDD = monoclonal immunoglobulin deposition disease; AL = light chain amyloid. *Representative references provided.
